# Perception of speaker sincerity in complex social interactions by cochlear implant users

**DOI:** 10.1371/journal.pone.0269652

**Published:** 2022-06-08

**Authors:** Kathrin Rothermich, Susannah Dixon, Marti Weiner, Madison Capps, Lixue Dong, Sébastien Paquette, Ning Zhou

**Affiliations:** 1 Department of Communication Sciences and Disorders, East Carolina University, Greenville, NC, United States of America; 2 Department of Psychology, University of Montréal, Montréal, QC, Canada; University of California Los Angeles, UNITED STATES

## Abstract

Understanding insincere language (sarcasm and teasing) is a fundamental part of communication and crucial for maintaining social relationships. This can be a challenging task for cochlear implant (CIs) users who receive degraded suprasegmental information important for perceiving a speaker’s attitude. We measured the perception of speaker sincerity (literal positive, literal negative, sarcasm, and teasing) in 16 adults with CIs using an established video inventory. Participants were presented with audio-only and audio-visual social interactions between two people with and without supporting verbal context. They were instructed to describe the content of the conversation and answer whether the speakers meant what they said. Results showed that subjects could not always identify speaker sincerity, even when the content of the conversation was perfectly understood. This deficit was greater for perceiving insincere relative to sincere utterances. Performance improved when additional visual cues or verbal context cues were provided. Subjects who were better at perceiving the content of the interactions in the audio-only condition benefited more from having additional visual cues for judging the speaker’s sincerity, suggesting that the two modalities compete for cognitive recourses. Perception of content also did not correlate with perception of speaker sincerity, suggesting that what was said vs. how it was said were perceived using unrelated segmental versus suprasegmental cues. Our results further showed that subjects who had access to lower-order resolved harmonic information provided by hearing aids in the contralateral ear identified speaker sincerity better than those who used implants alone. These results suggest that measuring speech recognition alone in CI users does not fully describe the outcome. Our findings stress the importance of measuring social communication functions in people with CIs.

## Introduction

Everyday social interactions are crucial for emotional well-being and maintaining relationships. To convey communicative messages, speakers use an array of verbal and nonverbal cues, such as discourse context, prosody, and facial expressions, to support language comprehension. However, sometimes these cues are inconsistent with each other, for example, when a speaker communicates sarcasm. As a form of irony, sarcasm is an important element of daily communication and can serve many social functions, including criticizing, saving face, and being humorous [1]. An example for sarcasm is saying “Oh I love it.” to a friend who cooked a meal when not enjoying it. Similarly, we use ironic forms of language such as teasing to enhance the affiliation between communication partners [2]. An example for teasing is saying “Oh I hate it.” to a friend who cooked a meal when enjoying it.

It is widely accepted that prosody, especially pitch, is a major indication of ironic statements [3, 4]. A few studies identified speech duration and rate, pitch variability, and absolute pitch height as acoustic parameters used to mark ironic statements [5, 6]. Among the various acoustic markers used to signal sarcasm, one of the primary cues is reduced pitch [6] as well as increased reduced pitch variability [7]. While there is less research on the acoustic cues of teasing, it is often signaled by laughter [8, 9]. However, it has been noted that there may not be a stereotypical “ironic tone of voice” because irony can take many different shapes [10]. This aligns with assumptions that an ironic meaning is often communicated through an incongruence of a statement and the context [11], and that acoustic markers of irony vary across individuals [7].

When prosodic cues are degraded, such as listening with an auditory prosthesis, understanding ironic statements can be challenging. Cochlear implants (CIs) are successful neural prostheses that restore hearing in profoundly deaf individuals, but the signal conveyed by the implant to the auditory nerve is severely degraded compared to acoustic hearing. Frequency resolution, which is crucial for perceiving pitch information, is limited not only by the small number of frequency bands available with the device but also by channel interactions [12]. Studies have shown that a small proportion of CI users maintain a somewhat typical place pitch (pitch-electrode) map consistent with the Greenwood function, and the remaining of the population shows either flat or non-monotonic growth of pitch on the tonotopic axis [13]. Pitch transmitted temporally is not readily available for CI users either, as CI users generally do not perceive pitch change as stimulation rate increases beyond 300 pulses a second, known as the “upper limit of pitch” [14–16]. Due to lack of salient pitch information (a primary cue for prosody), CI users are biased toward using less salient pitch cues [17], changes in amplitude or tempo (e.g., duration, speech rate, rhythm) and spectro-temporal (e.g., timbre) variations [18–20]. They often infer emotion from verbal cues rather than speech prosody [21]. Normal hearing adults listening to spectrally degraded speech simulating CIs are found to shift their attention to intensity and duration cues that co-vary in pitch, similarly to CI listeners [22]. CI users’ performance in identifying simple emotions in speech such as “angry” and “happy” is well below their normal-hearing peers [23]. This emotion perception deficit can extend beyond the realms of speech and can be observed for short emotional vocalizations [24], music [20, 25] and emotions conveyed via facial expressions [26].

Perception of emotional speech and irony remains an understudied area for CI users. The major body of the literature is limited to the perception of basic emotions, e.g., happy and angry [e.g., 17, 23], or focuses largely on the pediatric population [e.g., 27–29]. The present study examined adult CI users and measured their perception of speaker sincerity in complex social exchanges. We used stimuli selected from a validated video database designed specifically for studying ironic language, where two actors engage in various conversations and convey sincere or insincere speaker intentions [9]. The CI subjects were presented with audio-only and audio-visual versions of the stimuli. The stimuli were also manipulated to provide verbal context or no context cue to the speakers’ intention (see Table 2 for stimuli). Subjects were evaluated for their perception of the content of the conversation (i.e., what did the actors say?) and then their perception of the underlying speaker intentions (i.e., did they mean what they said?). Below we state our hypotheses on how these manipulated variables might affect perception.

First, we hypothesized that for CI users, the deficit of perception of speaker intentions would be particularly salient for the perception of insincere (sarcasm, teasing) relative to sincere intentions. Besides the “truth bias”, where we often assume that speakers are telling the truth [30], the insincere intentions may be particularly difficult for CI users. They must decode the possibly more complex and variable prosody in the insincere utterances to determine if they are incongruent with content [6, 7]. The difficulties with insincere intentions may be reduced by the availability of visual or context cues. The effect of visual information on auditory perception has been well established [e.g., 31, 32]. Most and Aviner [33] demonstrated that when auditory and visual information are congruent, performance in the audio-visual condition was better than audio-only but quite comparable to visual-alone condition among adolescent CI users. This suggests that visual cues were dominant in these tasks when the auditory cues are less salient [33]. Fengler and colleagues [34] provided further evidence of visual dominance, reporting greater interference of incongruent facial expressions in congenitally deafened CI users than the control group. For our tasks, the subjects might use different visual cues, i.e., lip reading or observing facial expressions, for perceiving content and speaker sincerity. Still, we hypothesized that performance would be better with additional visual information for both tasks. Contextual cues are especially important for comprehending ironic statements [35] and may reduce subjects’ response time to insincere utterances [36]. We hypothesized that discourse context would greatly facilitate performance, particularly for insincere intentions and when stimuli are presented without visual cues.

Lastly, the measured behavioral results were correlated with key demographic variables (e.g., duration of hearing loss) and device features. Since duration of deafness has been consistently identified to predict CI outcomes [37, 38], we hypothesized that perception in the audio-only condition would depend on subjects’ duration of hearing deprivation. We also hypothesized that CI users who have residual acoustic hearing in the contralateral ear and take advantage of pitch information provided by a hearing aid would perform better than those *without* access to acoustic hearing.

## Materials and methods

### Subjects and hardware

Sixteen cochlear implant users participated in the study. Six subjects were sequentially bilaterally implanted and had no residual hearing in either implanted ear. Five subjects were bimodal users, wearing a cochlear implant on one side and a hearing aid on the contralateral side. All participants were adult, post-lingually deafened, native English-speaking users of either Cochlear© (Cochlear Corporation, Englewood, CO) or Advanced Bionics (Advanced Bionics, Valencia, CA) devices. TH16 had early-onset perilingual hearing loss and became proudly deaf when he was an adult. Subjects’ mean age at the time of testing was 68.50 years, the mean duration of hearing loss was 33.29 years, and the mean CI experience was 8.49 years. Demographic information for participants and test ears is shown in Table 1. Duration of hearing loss was defined in the study as the time between the onset of hearing loss and implantation. All subjects provided written informed consent before taking part in the study. This study was approved by the East Carolina University Institutional Review Board.

**Table 1:** Demographic information.

Subject	Ear	Gender	Age	CI use (yrs)	Hearing Loss Duration	Implant Type	Processor Type	Speech processing strategy	Device type
DB1	L	M	80.35	17.27	13.06	CI24R (CS)	CP910	ACE	Bilateral CI
DB1	R	M	80.35	11.30	19.04	CI24RE (CA)	CP810	ACE	Bilateral CI
CJ3	L	F	68.99	12.98	15.35	CI24RE (CA)	CP920	ACE	Bilateral CI
CJ3	R	F	68.99	14.50	13.83	CI24RE (CA)	CP920	ACE	Bilateral CI
NM4	L	F	60.15	7.77	22.38	CI24RE (CA)	CP810	ACE	Unilateral CI
SG7	R	F	73.69	8.50	34.83	CR24RE (CA)	CP810	ACE	Bimodal
TH16	L	M	57.69	12.60	45.09	CI24RE (CA)	CP810	ACE	Bilateral CI
TH16	R	M	57.69	10.68	47.01	CI24RE (CA)	CP810	ACE	Bilateral CI
SP18	L	F	67.43	4.71	33.63	CI422	CP910	ACE	Bimodal
FP19	L	F	72.64	12.00	44.33	CI24RE (CA)	CP1000	ACE	Unilateral CI
DA22	R	F	74.47	6.94	17.39	CI4RE (CA)	CP920	ACE	Unilateral CI
KS25	L	F	62.18	11.67	18.67	CI24RE (CA)	CP900	ACE	Bilateral CI
KS25	R	F	62.18	10.92	19.42	CI24RE (CA)	CP900	ACE	Bilateral CI
DK27	L	M	59.76	12.33	42.43	CI512	CP920	ACE	Bilateral CI
DK27	R	M	59.76	13.33	41.43	CI24RE (CA)	CP920	ACE	Bilateral CI
KK28	R	F	76.64	12.58	58.06	HR90K	Naida CI Q70	HiRes Optima-S	Unilateral CI
CP30	L	F	60.77	6.91	53.86	HiFocus 1J	Naida CI Q90	HiRes Optima-S	Bilateral CI
CP30	R	F	60.77	12.68	48.09	HiFocus 1J	Naida CI Q90	HiRes Optima-S	Bilateral CI
SS31	L	M	69.71	3.82	26.51	CI422	Kanso	ACE	Unilateral CI
RS32	L	M	68.88	2.51	47.36	HiFocus ms	Naida CI Q90	HiRes Optima-S	Bimodal
PV33	R	F	68.93	2.13	62.20	HiFocus 1J	Naida CI Q90	HiRes Optima Paired	Unilateral CI
BB34	L	F	72.68	1.59	14.74	HiFocus ms	Naida CI Q90	HiRes Optima-S	Bimodal
BB35	L	M	77.66	1.60	21.06	HiFocus ms	Naida CI Q90	HiRes Optima-P	Bimodal

### Stimuli

The stimuli were chosen from a validated video inventory for testing social language perception [9]. The inventory has been previously used in studies with young adults [39], typically developing children [40], and older adults [41]. It consists of short video recordings depicting social exchanges intended to be sincere (positive and negative), and insincere (sarcasm and teasing). The videos in the complete published RISC inventory had been validated previously with 31 adult participants (mean age = 23.21 years, SD = 3.88). For the specific subset of 96 videos used in the current study, young typical-hearing adults in the study by Rothermich and Pell [9] identified the speaker’s intention with an average accuracy of 84.26% correct (literal positive: M = 87%, SD = 17%, literal negative: M = 96%, SD = 3%, sarcastic: M = 76%, SD = 24%, teasing: M = 89%, SD = 4%). The different attitudes in the videos are expressed by using prosodic cues, facial expressions, and body language.

We have described the acoustic properties of the stimuli in more detail in a previous publication [see Table 2 in 40]. The stimuli used for the present study were selected from these videos based on the criterion that they should be comprehensible in the audio-only version. The selected stimuli consisted of videos recorded from 24 scenes x 4 intentions (94 trials in total) depicting a couple (a female person and a male person) having a conversation. In each scene, the final statement is produced with different intonations and visual expressions to convey four different intentions (literal positive, literal negative, sarcasm, and teasing). Of the 24 scenes, verbal context was provided in 15 scenes. Example scenes are shown in Table 2. Each of the 96 videos was transcribed, and the transcriptions were used to compare to the subjects’ responses (see details under Procedures). Sounds of the 96 videos were extracted and processed to have equal RMS (root mean square) values in MATLAB. The stimuli were presented in audio-only and audio-visual conditions; for a total of 192 stimuli: 4 intentions × 24 scenes × 2 presentation modes.

**Table 2:** Example scenes used in the experiment.

Scene “Wedding” (with verbal context)		Scene “Party” (no verbal context)	
Literal positive	Lisa on the phone:… I’m looking forward already! I’ll call you later!	Literal positive	Lisa: Do you think the party was a success?
	Paul: Are you gonna come with me to Sarah’s wedding?		Paul: Yeah, I had a great time!
	Lisa: Yeah, it is gonna be fun!		
Literal negative	Lisa on the phone:… and I don’t really wanna go there, anyways. I’ll call you later!	Literal negative	Lisa: Do you think the party was a success?
	Paul: Are you gonna come with me to Sarah’s wedding?		Paul: No, no one had fun.
	Lisa: No, weddings aren’t really my thing.		
Sarcasm	Lisa on the phone:… and I don’t really wanna go there, anyways. I’ll call you later!	Sarcasm	Lisa: Do you think the party was a success?
	Paul: Are you gonna come with me to Sarah’s wedding?		Paul (sarcastic): Yeah, I had a great time!
	Lisa (sarcastic): Yeah, it is gonna be fun!		
Teasing	Lisa on the phone:… I’m looking forward already! I’ll call you later!	Teasing	Lisa: Do you think the party was a success?
	Paul: Are you gonna come with me to Sarah’s wedding?		Paul: No, no one had fun (laughs).
	Lisa: No, weddings aren’t really my thing (laughs).		

### Procedures

Subjects were seated in a sound-attenuated booth. The audio signals were delivered from a loudspeaker placed 1 meter from the head of the subject at 0 azimuths at 65 dB (A). The videos were displayed on a widescreen monitor placed right below the speaker. The bilaterally implanted subjects used both of their processors during the experiment. The bimodal subjects used both their implant and hearing aid (on the side contralateral to the implant) during the experiment. The rationale for allowing the subjects to use both ears, rather than testing just the implanted ear or testing a single implanted ear, was to mimic real-life situations, where the subjects would use all devices available for such social interactions. MATLAB was used to create a user interface for delivering the test and collecting subjects’ responses.

The stimuli were presented in two blocks (audio-only and audio-visual). The audio-only condition was always tested first, followed by the audio-visual condition. The audio-visual condition was presented second because this condition was expected to be easier for the subjects, especially those who lip-read. Thus, the visual context was provided in a second block to avoid over-familiarization with the stimuli. Within each block, the stimuli were fully randomized. Different randomizations were used if the first audio-visual stimulus was the same as the last audio-only stimulus. Each stimulus was presented to the subject as many times as needed. We acknowledge that this does not mimic a real-life situation, but stimuli were presented multiple times to avoid a floor effect. When the subjects were ready, they first described the conversation content in their own words to the best of their ability, i.e., what did they say? The verbal description was typed either by the subject or by the experimenter, and the response was saved for offline analysis. After describing the conversation’s content, the subject was then asked about the speaker’s intention, i.e., did they mean what they said? The answer options were “Yes” and “No”; “Yes” would be a correct response if the intentions were sincere (literal positive or negative). “No” would be a correct response if the intentions were teasing or sarcasm. Subjects were encouraged to take frequent breaks during the experiment. The average testing time was 6 hours, depending on how often the stimuli were repeated.

Offline, the subject’s description of the conversation content was compared to the transcript of each stimulus. Three raters independently rated the subject’s description as correct or incorrect. The response was entered into the analysis of speakers’ intention only if at least two raters agreed that the subject understood the conversation content. This was based on the assumption that it would be impossible for subjects to perceive, even with visual cues, the intention underlying such complex social exchanges if they did not understand the content in the first place.

## Results

**Fig 1:**
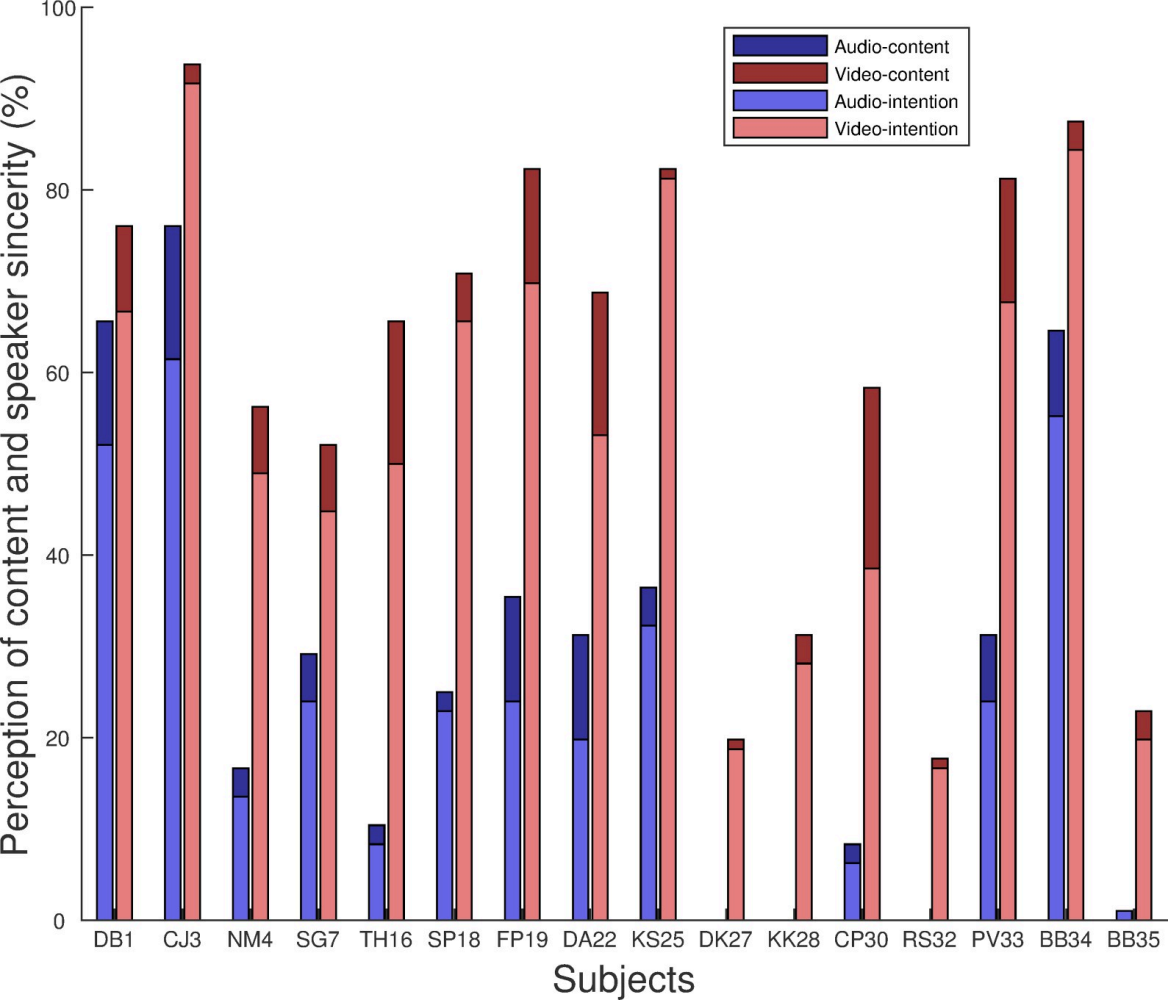
Performance for individual subjects. Blue bars represent performance in the audio-only condition. The red bars represent the audio-visual condition. The bars represent the percentages of correctly perceived conversations in terms of the content question (dark-colored bars) and identifying speaker sincerity (light-colored bars) relative to the total number of stimuli.

**Fig 2:**
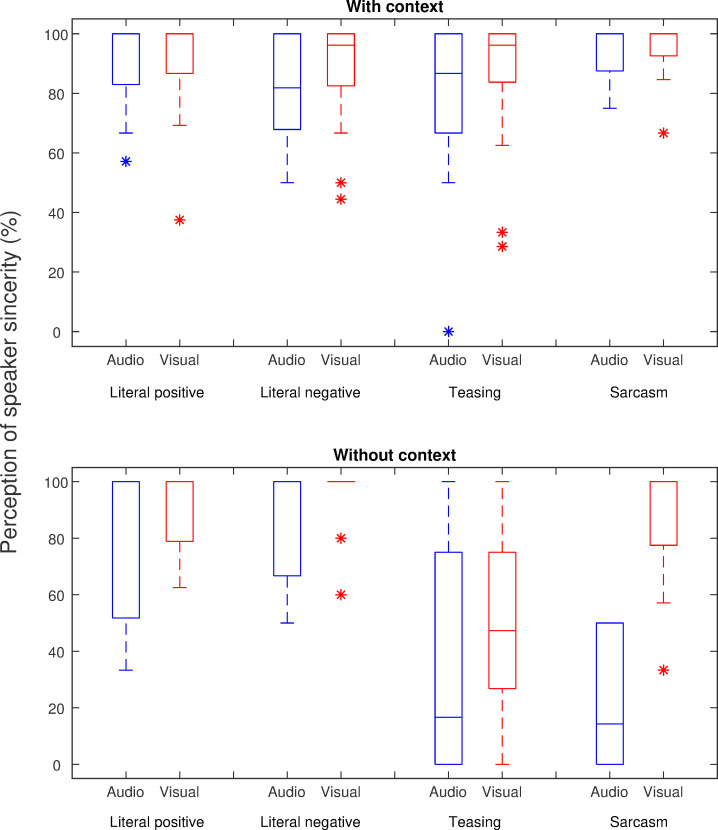
Box plot of perception of speaker’s intentions collapsed across subjects. The top panel shows performance with context, and the bottom panel shows performance without context. Percent correct scores were calculated based on the number of correctly perceived intentions relative to the number of correctly perceived contents. Bars represent group medium. Asterisks show outliers.

The dark-colored bars (dark red and blue) in Fig 1 show the percentage of conversations (out of the 96 stimuli) that the subjects perceived correctly in terms of the content question. The light-colored bars in the figure show the percentage of conversations (out of the 96 stimuli) that the subjects perceived correctly in terms of both the content and the underlying speaker sincerity. Perception of content significantly improved in the audio-visual condition relative to the audio-only condition [t (14) = 9.26, p < 0.001] (red versus blue bars). Paired T-tests showed that for both the audio-only and audio-visual conditions, perception of speaker sincerity significantly dropped from perception of content, indicating a deficit for perception of speaker sincerity [audio-only: t (12) = -5.04, p < 0.001; audio-visual: t (15) = -9.46, p = 0.001]. Perception of speaker sincerity was then quantified as the number of correctly perceived sincerities relative to the number of correctly perceived contents (Fig 2). The percentages inform performance at extracting speaker sincerity given the content was perfectly understood. Pearson’s correlations suggest that perception of speaker sincerity was not correlated with perception of content under either audio-only [r = -0.16, p = 0.61] or audio-visual conditions [r = 0. 021, p = 0.96].

Main effects of speaker intention categories (literal positive, literal negative, teasing, and sarcasm), presentation mode (audio-only vs. audio-visual), and availability of context on perceiving speaker intentions were examined. To keep the subjects with missing data in the analysis, a mixed linear model was used. Results of the analysis showed that subjects performed differently across intention categories [F (3, 72) = 9.04, p < 0.001]. Performance was better in the audio-visual condition than audio alone [F (1, 72) = 7.46, p = 0.008]. Performance was better with context than without [F (1, 93) = 22.34, p < 0.001]. Performance for stimuli with context was not correlated with performance without context. [Audio: r = 0.01, p = 0.85; Audio-visual: r = 0.42, p = 0.11]. The benefit of visual information was greater for stimuli without context than with context [presentation mode × context: F (1, 80) = 4.82, p = 0.03]. Performance differences in intention categories were also greater without context than with context [intention × context: F (3, 78) = 8.87, p < 0.001]. Because the benefit of visual information was the same across intention categories [intention × presentation mode: F (3, 80) = 0.25, p = 0.87], data were collapsed across presentation mode to examine the effect of sincerity on perception. With context, performance was better for sarcasm than literal negative [t (15) = -3.22, p = 0.005]; all other comparisons were non-significant (all p > 0.05). Without context, performance for the two sincere intentions was the same [t (15) = -0.87, p = 0.39]; performance for sarcasm was better than teasing [t (15) = -2.62, p = 0.02]. Comparing performance between sincere and insincere intentions, literal positive was better than teasing [t (15) = 4.92, p < 0.001], and sarcasm [t (15) = 2.22, p = 0.03]; literal negative was also better than teasing [t (15) = -2.62, p = 0.02] and sarcasm [t (15) = 4.05, p = 0.001].

**Fig 3:**
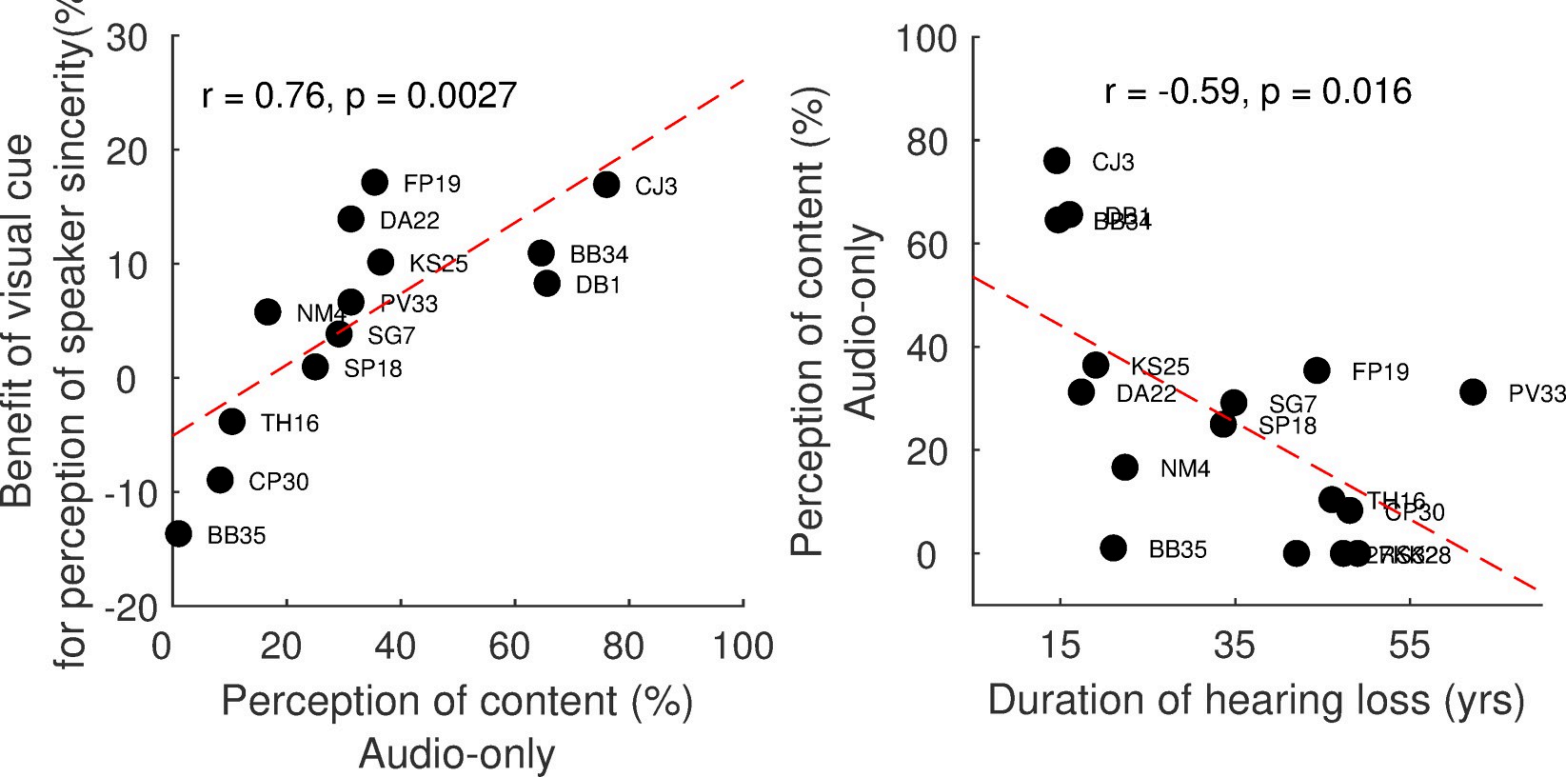
Left panel: The relationship between the advantage of visual cues in identifying speakers’ sincerity and perception of content in the audio-only condition. Right panel: Correlation between the perception of content in the audio-only condition and duration of hearing loss.

The benefit of visual cues for the perception of speaker intentions collapsed across conditions was correlated with subjects’ performance in the perception of content in the audio-only condition (r = 0.76, p = 0.003) (Fig 3, left panel). The benefit of visual cues was quantified by calculating the difference in performance between the audio-visual and audio-only conditions. The additional visual information was more likely to help subjects with perceiving speaker sincerity if they did not struggle with understanding the audio-only stimuli. Further, no relationship was found between the benefit of visual cues for understanding the content and that for understanding speaker’s sincerity [r = 0.17, p = 0.58].

Next, the effect of device features on the perception of content and speaker sincerity was analyzed. Subjects differed in their device types, i.e., unilaterally implanted, bilaterally implanted, and bimodal users. A one-way repeated-measures ANOVA showed no significant effect of device type on perception of content in the audio-only condition (F (2,15) = 0.25, p = 0.79). However, a significant effect of device type was found on the perception of speakers’ sincerity in the audio-only condition [F (2,12) = 6.42, p = 0.02]. Post-hoc analysis further showed that the bimodal subjects outperformed both the unilaterally and bilaterally implanted subjects (one-tailed, all p < 0.05). When examining just the data without context, the device effect disappeared partially because this challenging listening condition resulted in a much smaller sample size due to missing data [F (2,12) = 0.92, p = 0.43].

The relationship between performance and the subjects’ demographic variables was weak. Longer duration of hearing loss was associated with worse perception of content in the audio-only condition [r = -0.60, p = 0.02] (Fig 3, right panel). No other demographic variables, such as age and duration of CI use, were predictive of perception of content or speaker sincerity (all p > 0.05).

## Discussion

The current study examined adult CI users’ perception of speaker sincerity in complex social interactions. Our results indicated a deficit, in that even when the CI users understood the content of the conversation perfectly; they were not always able to extract the underlying speaker sincerity. The deficit was more salient for identifying insincere versus sincere intentions or when visual cues or verbal context cues were absent. Visual information was more likely to help those who did not struggle with the content of the conversations, which could suggest a competition of cognitive resources. A shorter duration of hearing loss helped subjects understand the content, while access to pitch information via acoustic hearing benefited their perception of speaker sincerity. Below we provide a more detailed discussion of our findings.

### Relationship between perception of content and speaker sincerity

It is clear from **Fig 1** that subjects who understood the interactions in terms of the content did not always correctly perceive the speaker’s sincerity, confirming a deficit in CI users of their ability to understand social aspects of language. More importantly, perception of content and speaker sincerity was not correlated. These data suggest that the acoustic cues required for understanding segmental speech are different from those for understanding the suprasegmental cues that signal a speakers’ sincerity. Further evidence came from the lack of correlation between perception of speaker sincerity with verbal (segmental) context and perception of speaker sincerity without context. For the latter, perception would depend solely on speech prosody. Thus, the subjects’ access and utility of the two sets of cues may not always be linked. These results suggest that measuring segmental speech perception in CI users may not fully describe the implant’s efficacy for providing social communication for its users.

### The effect of visual information

Our results revealed that the addition of visual information facilitated comprehension of the content with a large effect size. The large effect size could partially result from an order effect because the audio-visual condition was always presented after the audio-only condition. For perceiving speaker sincerity, the benefit of visual cues was consistent with previous reports [34], but the effect was smaller for stimuli with verbal context than without. The results could suggest that the CI subjects put a greater weight on the verbal context cues than the visual cues if the verbal context was available. Visual information may be redundant if a given context is supportive enough for a literal or nonliteral interpretation of an utterance. However, examining the data in Fig 1 (upper panel), the smaller visual effect could also be due to a ceiling effect, where the performance with verbal context in the audio alone condition was already rather good. In future studies, introducing incongruent visual and context cues could be used to determine the exact weighting between these cues.

The mechanism underlying the visual effect for the perception of content and speaker sincerity might be different. Looking at the speaker’s face may have helped the subjects understand the content via lip-reading. In contrast, for the perception of speaker sincerity, subjects might observe the speakers’ facial expressions and subtle body gestures. The fact that there was no correlation between the effect of visual cues for understanding content and speaker sincerity supports the idea that the listeners were using different visual cues for the two tasks. Further, our results showed that additional visual information tended to help those who did not struggle with understanding the content of the conversation in the audio-only condition (**Fig 3**, left panel). In the present tasks, the subjects must first understand the content of the conversation to be able to answer the question if the speaker meant what they said. We speculate less auditory listening effort used to understand the content might free up cognitive resources with which the visual cues can be processed and ultimately be used to identify sincerity. A similar suggestion has been put forward by Chatterjee and colleagues [42]; that obligatory speech perception might take away cognitive resources from more complex tasks such as emotion recognition.

### Effect of speaker intention category

Our results also indicated that the subjects identified sincere intentions better than teasing and sarcasm. The effect was more prominent when the stimulus did not provide a verbal context. One reason for this finding could be a so-called “truth bias” [30]. It posits that by default, we assume that conversation partners tell the truth, i.e., that they mean what they said. Therefore, it is possible that subjects were biased to believe that the speaker is being sincere since that represents the unmarked intention. The incongruent/ambiguous nature of sarcasm and teasing statements could present a challenge for listeners generally. The difficulties could also be due to acoustic differences between the stimuli in that prosody in the insincere utterances was more complex and variable. Nonetheless, unlike basic emotions such as “happy” and ‘‘sad”, it may be difficult to quantify these acoustic differences due to a lack of a stereotyped “ironic voice”. Of all intentions, teasing was the least correctly identified. This was in line with previous results in that teasing is harder to infer during social communication compared with sarcasm [39, 43]. We attribute this to several factors. It could be the frequency in daily life—sarcasm occurs more frequently than teasing and is recognized faster and with higher accuracy [44–46]. This dichotomy between sarcasm and teasing perception is often referred to as an “asymmetry of affect” [45] between these two types of irony. It indicates that while sarcasm still alludes to social norms of politeness by using positive language, teasing is riskier since it does not adhere to these norms on the surface level [47]; thus, it is harder to recognize.

### Demographic variables

The participants in the present study used different device configurations, i.e., some were bilaterally implanted; some were unilaterally implanted and completely deaf in the contralateral ear; the rest of the participants were unilaterally implanted, had residual acoustic hearing in the contralateral ear, and used a hearing aid. All participants used their hearing devices during the tasks, as they would in a real-life listening situation. The most interesting yet somewhat anticipated results were that the three groups performed the same regardless of their device type for the perception of content. However, the bimodal users outperformed the other two groups and benefited from using their hearing aids to perceive speaker sincerity. Note that these effects were measured in the audio-only conditions. We could not confirm the device’s effect on performance for just the stimuli without context. Four subjects could not perform the task (0% on content), greatly reducing the sample size for comparing three groups. Generally, the data provided evidence that the acoustic cues for perceiving segmental (content) versus suprasegmental (sincerity) speech do not overlap considerably and a hearing aid provides better access to the suprasegmental information. There is substantial evidence to indicate that amplified acoustic information, combined with electrical stimulation, consistently improves CI users’ pitch perception. Even if acoustic information is often spectrally smeared and mismatched with what is provided by the implant, this benefit has been consistently demonstrated in lexical tone perception [48, 49], music perception [50], and perception of competing speech [51, 52].

Subjects’ duration of hearing deprivation before implantation was associated with their performance in the content task in the audio-only condition, but not predictive of their perception of the sincerity or perception in the audio-visual conditions. These findings suggest that hearing deprivation before implantation may not be a strong factor driving individual variances in speech prosody perception but plays an important role in the perception of segmental information. Lastly, our subject sample were older adults. It is possible that the declining cognitive functions have contributed to performance. Further studies are warranted to investigate the interactions between the factors of aging, cognitive function, hearing devices on prosody processing using a neural prosthesis.

## Conclusion

Our data suggest that CI users’ ability to perceive speaker sincerity is impaired and this impairment is not related to their performance in understanding segmental speech or the content of the conversations. Such deficit may be alleviated if the speakers provided a verbal context to their ironic statement or provided nonverbal body language cues. The deficit may also be alleviated if listeners used a bimodal system where the hearing aid provided resolved pitch information. Our data suggest that evaluating CI outcomes using only speech perception measures does not fully describe their ability for social communication. The outcomes will inform new directions in rehabilitation schemes that enable CI listeners to capitalize on multimodal cues and the combination of acoustic and electric stimulation to optimize social communication with the device.

## References

[1] Dews S, Kaplan J, Winner E. Why not say it directly? The social functions of irony. Discourse Process. 1995;19: 347–367.

[2] Seckman MA, Couch CJ. Jocularity, sarcasm, and relationships: An empirical study. J Contemp Ethnogr. 1989;18: 327–344.

[3] Anolli L, Ciceri R, Infantino MG. From “blame by praise” to “praise by blame”: Analysis of vocal patterns in ironic communication. Int J Psychol. 2002;37: 266–276. doi: 10.1080/00207590244000106

[4] Attardo S, Eisterhold J, Hay J, Poggi I. Multimodal markers of irony and sarcasm. Humor—Int J Humor Res. 2003;16. doi: 10.1515/humr.2003.012

[5] Bryant GA. Prosodic Contrasts in Ironic Speech. Discourse Process. 2010;47: 545–566. doi: 10.1080/01638530903531972

[6] Cheang HS, Pell MD. The sound of sarcasm. Speech Commun. 2008;50: 366–381. doi: 10.1016/j.specom.2007.11.003

[7] Mauchand M, Vergis N, Pell M. Ironic tones of voices. 9th International Conference on Speech Prosody 2018. ISCA; 2018. pp. 443–447. doi: 10.21437/SpeechProsody.2018-90

[8] Gibbs RW. Irony in Talk Among Friends. Metaphor Symb. 2000;15: 5–27. doi: 10.1080/10926488.2000.9678862

[9] Rothermich K, Pell MD. Introducing RISC: A New Video Inventory for Testing Social Perception. Filik R, editor. PLOS ONE. 2015;10: e0133902. doi: 10.1371/journal.pone.0133902 26226009 PMC4520563

[10] Bryant GA, Fox Tree JE. Is there an Ironic Tone of Voice? Lang Speech. 2005;48: 257–277. doi: 10.1177/00238309050480030101 16416937

[11] Katz AN, Lee CJ. The role of authorial intent in determining verbal irony and metaphor. Metaphor Symb. 1993;8: 257–279.

[12] Nelson DA, Kreft HA, Anderson ES, Donaldson GS. Spatial tuning curves from apical, middle, and basal electrodes in cochlear implant users. J Acoust Soc Am. 2011;129: 3916–3933. doi: 10.1121/1.3583503 21682414 PMC3135148

[13] Vermeire K, Nobbe A, Schleich P, Nopp P, Voormolen MH, Van de Heyning PH. Neural tonotopy in cochlear implants: an evaluation in unilateral cochlear implant patients with unilateral deafness and tinnitus. Hear Res. 2008;245: 98–106. doi: 10.1016/j.heares.2008.09.003 18817861

[14] Baumann U, Nobbe A. Pulse rate discrimination with deeply inserted electrode arrays. Hear Res. 2004;196: 49–57. doi: 10.1016/j.heares.2004.06.008 15464301

[15] Kong Y-Y, Carlyon RP. Temporal pitch perception at high rates in cochlear implants. J Acoust Soc Am. 2010;127: 3114–3123. doi: 10.1121/1.3372713 21117760

[16] Zhou N, Mathews J, Dong L. Pulse-rate discrimination deficit in cochlear implant users: is the upper limit of pitch peripheral or central? Hear Res. 2019;371: 1–10. doi: 10.1016/j.heares.2018.10.018 30423498 PMC6309496

[17] Gilbers S, Fuller C, Gilbers D, Broersma M, Goudbeek M, Free R, et al. Normal-hearing listeners’ and cochlear implant users’ perception of pitch cues in emotional speech. -Percept. 2015;6: 0301006615599139. doi: 10.1177/0301006615599139 27648210 PMC5016815

[18] Gfeller K, Witt S, Mehr MA, Woodworth G, Knutson J. Effects of frequency, instrumental family, and cochlear implant type on timbre recognition and appraisal. Ann Otol Rhinol Laryngol. 2002;111: 349–356. doi: 10.1177/000348940211100412 11991588

[19] Looi V, King J, Kelly-Campbell R. A music appreciation training program developed for clinical application with cochlear implant recipients and hearing aid users. Seminars in Hearing. Thieme Medical Publishers; 2012. pp. 361–380.

[20] Paquette S, Ahmed GD, Goffi-Gomez MV, Hoshino ACH, Peretz I, Lehmann A. Musical and vocal emotion perception for cochlear implants users. Hear Res. 2018;370: 272–282. doi: 10.1016/j.heares.2018.08.009 30181063

[21] Richter ME, Chatterjee M. Weighting of prosodic and lexical-semantic cues for emotion identification in spectrally-degraded speech and with cochlear implants. Ear Hear. 2021;42: 1727–1740. doi: 10.1097/AUD.0000000000001057 34294630 PMC8545870

[22] Peng S-C, Chatterjee M, Lu N. Acoustic Cue Integration in Speech Intonation Recognition With Cochlear Implants. Trends Amplif. 2012;16: 67–82. doi: 10.1177/1084713812451159 22790392 PMC3560417

[23] Luo X, Fu Q-J, Galvin JJ. Cochlear Implants Special Issue Article: Vocal Emotion Recognition by Normal-Hearing Listeners and Cochlear Implant Users. Trends Amplif. 2007;11: 301–315. doi: 10.1177/1084713807305301 18003871 PMC4111530

[24] Deroche ML, Felezeu M, Paquette S, Zeitouni A, Lehmann A. Neurophysiological differences in emotional processing by cochlear implant users, extending beyond the realm of speech. Ear Hear. 2019;40: 1197–1209. doi: 10.1097/AUD.0000000000000701 30762600

[25] Ambert-Dahan E, Giraud A-L, Sterkers O, Samson S. Judgment of musical emotions after cochlear implantation in adults with progressive deafness. Front Psychol. 2015;6. Available: https://www.frontiersin.org/article/10.3389/fpsyg.2015.00181 25814961 10.3389/fpsyg.2015.00181 PMC4357245

[26] Ambert-Dahan E, Giraud A-L, Mecheri H, Sterkers O, Isabelle M, Samson S. Emotional recognition of dynamic facial expressions before and after cochlear implantation in adults with progressive deafness. Semin Hear. 2017;354: 64:72. 10.1016/j.heares.2017.08.007 28886405

[27] Hopyan-Misakyan TM, Gordon KA, Dennis M, Papsin BC. Recognition of affective speech prosody and facial affect in deaf children with unilateral right cochlear implants. Child Neuropsychol. 2009;15: 136–146. doi: 10.1080/09297040802403682 18828045

[28] Panzeri F, Cavicchiolo S, Giustolisi B, Di Berardino F, Ajmone PF, Vizziello P, et al. Irony Comprehension in Children With Cochlear Implants: The Role of Language Competence, Theory of Mind, and Prosody Recognition. J Speech Lang Hear Res. 2021;64: 3212–3229. doi: 10.1044/2021_JSLHR-20-00671 34284611

[29] Wiefferink CH, Rieffe C, Ketelaar L, De Raeve L, Frijns JH. Emotion understanding in deaf children with a cochlear implant. J Deaf Stud Deaf Educ. 2013;18: 175–186. doi: 10.1093/deafed/ens042 23232770

[30] Levine TR. Truth-Default Theory (TDT): A Theory of Human Deception and Deception Detection. J Lang Soc Psychol. 2014;33: 378–392. doi: 10.1177/0261927X14535916

[31] McGurk H, MacDonald J. Hearing lips and seeing voices. Nature. 1976;264: 746–748. doi: 10.1038/264746a0 1012311

[32] Munhall KG, Jones JA, Callan DE, Kuratate T, Vatikiotis-Bateson E. Visual Prosody and Speech Intelligibility: Head Movement Improves Auditory Speech Perception. Psychol Sci. 2004;15: 133–137. doi: 10.1111/j.0963-7214.2004.01502010.x 14738521

[33] Most T, Aviner C. Auditory, visual, and auditory–visual perception of emotions by individuals with cochlear implants, hearing aids, and normal hearing. J Deaf Stud Deaf Educ. 2009;14: 449–464. doi: 10.1093/deafed/enp007 19398533

[34] Fengler I, Nava E, Villwock AK, Büchner A, Lenarz T, Röder B. Multisensory emotion perception in congenitally, early, and late deaf CI users. PloS One. 2017;12: e0185821. doi: 10.1371/journal.pone.0185821 29023525 PMC5638301

[35] Kreuz RJ, Glucksberg S. How to be sarcastic: The echoic reminder theory of verbal irony. J Exp Psychol Gen. 1989;118: 374–386. doi: 10.1037//0096-3445.118.4.374

[36] Giora R, Fein O, Laadan D, Wolfson J, Zeituny M, Kidron R, et al. Expecting Irony: Context Versus Salience-Based Effects. Metaphor Symb. 2007;22: 119–146. doi: 10.1080/10926480701235346

[37] Rubinstein JT, Parkinson WS, Tyler RS, Gantz BJ. Residual speech recognition and cochlear implant performance: effects of implantation criteria. Am J Otol. 1999;20: 445–452. 10431885

[38] Zhou N, Zhu Z, Dong L, Galvin J. Sensitivity to Pulse Phase Duration as a Marker of Neural Health Across Cochlear Implant Recipients and Electrodes. J Assoc Res Otolaryngol. 2021;22: 177–192. doi: 10.1007/s10162-021-00784-5 33559041 PMC7943680

[39] Joergensen GH, Makarla PR, Fammartino M, Benson L, Rothermich K. No, No One Had Fun. Individual Differences in Nonliteral Language Perception. Lang Speech. 2021; 002383092110108. doi: 10.1177/00238309211010859 34148389

[40] Rothermich K, Caivano O, Knoll LJ, Talwar V. Do they really mean it? Children’s inference of speaker intentions and the role of age and gender. Lang Speech. 2020;63: 689–712. doi: 10.1177/0023830919878742 31631741

[41] Rothermich K, Giorio C, Falkins S, Leonard L, Roberts A. Nonliteral language processing across the lifespan. Acta Psychol (Amst). 2021;212: 103213. doi: 10.1016/j.actpsy.2020.103213 33220614

[42] Chatterjee M, Zion DJ, Deroche ML, Burianek BA, Limb CJ, Goren AP, et al. Voice emotion recognition by cochlear-implanted children and their normally-hearing peers. Hear Res. 2015;322: 151–162. doi: 10.1016/j.heares.2014.10.003 25448167 PMC4615700

[43] Rothermich K, Schoen Simmons E, Rao Makarla P, Benson L, Plyler E, Kim H, et al. Tracking nonliteral language processing using audiovisual scenarios. Can J Exp Psychol Can Psychol Expérimentale. 2021 [cited 7 Jun 2021]. doi: 10.1037/cep0000223 33793260

[44] Caillies S, Gobin P, Obert A, Terrien S, Coutté A, Iakimova G, et al. Asymmetry of affect in verbal irony understanding: What about the N400 and P600 components? J Neurolinguistics. 2019;51: 268–277. doi: 10.1016/j.jneuroling.2019.04.004

[45] Clark HH, Gerrig RJ. On the pretense theory of irony. J Exp Psychol Gen. 1984;113: 121–126. doi: 10.1037//0096-3445.113.1.121 6242407

[46] Matthews JK, Hancock JT, Dunham PJ. The roles of politeness and humor in the asymmetry of affect in verbal irony. Discourse Process. 2006;41: 3–24.

[47] Pexman PM, Zvaigzne MT. Does Irony Go Better With Friends? 2004; 37–41. doi: 10.1207/s15327868ms1902

[48] Tao D-D, Liu J-S, Yang Z-D, Wilson BS, Zhou N. Bilaterally combined electric and acoustic hearing in Mandarin-speaking listeners: The population with poor residual hearing. Trends Hear. 2018;22: 2331216518757892. doi: 10.1177/2331216518757892 29451107 PMC5818091

[49] Zhou Q, Bi J, Song H, Gu X, Liu B. Mandarin lexical tone recognition in bimodal cochlear implant users. Int J Audiol. 2020;59: 548–555. doi: 10.1080/14992027.2020.1719437 32302240

[50] Kong Y-Y, Stickney GS, Zeng F-G. Speech and melody recognition in binaurally combined acoustic and electric hearing. J Acoust Soc Am. 2005;117: 1351–1361. doi: 10.1121/1.1857526 15807023

[51] Dorman MF, Gifford RH, Spahr AJ, McKarns SA. The benefits of combining acoustic and electric stimulation for the recognition of speech, voice and melodies. Audiol Neurotol. 2008;13: 105–112. doi: 10.1159/000111782 18057874 PMC3559130

[52] Zhang T, Spahr AJ, Dorman MF, Saoji A. The relationship between auditory function of non-implanted ears and bimodal benefit. Ear Hear. 2013;34: 133. doi: 10.1097/AUD.0b013e31826709af 23075632 PMC3549325

